# Internal medicine at the crossroads of long COVID diagnosis and management

**DOI:** 10.3389/fmed.2025.1521472

**Published:** 2025-05-02

**Authors:** Brigitte Ranque, Elie Cogan

**Affiliations:** ^1^Service de médecine interne, Hôpital Européen Georges-Pompidou, Unité CASPER, Hôtel Dieu, AP-HP, Université Paris Cité, Paris, France; ^2^Département de médecine interne, Hôpital Delta (CHIREC), Université Libre de Bruxelles, Brussels, Belgium

**Keywords:** long COVID-19, functional somatic disorder (FSD), post-acute COVID-19 syndrome, internal medicine, holistic care

## Abstract

The lack of specificity in its definition is a major obstacle to both explanatory and therapeutic research in long COVID. It brings together, on the one hand, patients with severe COVID-19 who suffer the classic complications of prolonged hospitalization and decompensation of comorbidities and, on the other hand, patients with non-severe acute COVID-19 who report multiple symptoms that cannot be fully explained by a biomechanical model. Indeed, despite numerous studies, it remains unclear how persistent viral infection, immunological or coagulation disturbances may contribute mechanistically to long COVID. Nevertheless, internal medicine should be in good place to manage these patients. Indeed, the diversity of symptoms may evoke a broad spectrum of differential diagnoses that are familiar to internists. Their experience in the exploration of unexplained symptoms is also valuable. It can reduce the need for multiple consultations with specialists and unnecessary laboratory or imaging tests. However, long COVID diagnosis cannot be limited to the exclusion of all other conditions one by one. An open and non-dualistic approach is required to identify other mechanisms that may explain the symptoms. Based on their clinical experience, most French internists who responded to an opinion survey consider that long COVID corresponds most closely to a functional somatic disorder (FSD) and seek the help of specialists in mental health care to assist in the management of the patients in a multi-disciplinary approach. However, as with other FSDs, patients with long COVID are usually reluctant to be managed by mental health care specialists, given the very physical nature of their presentation. Unfortunately, most physicians are in turn reluctant to take care of them, due to poor knowledge about FSD, leading to management failure. Alternatively, a comprehensive multidisciplinary care orchestrated by an experienced internist is generally well-accepted. It includes providing rational cognitive explanations for the symptoms and support for behavioral changes tailored to the patient. While waiting for hypothetical randomized controlled trials assessing drugs with positive results, such a holistic approach has been successfully applied in many individuals with severe long COVID. However, its generalization would require a much broader training for FSD of all health care providers.

## Introduction

Long COVID is considered a public health problem, since its incidence was estimated as high as 10% among patients infected by SARS-CoV-2 (1). More recent data suggest a gradual reduction in the risk over time. The cumulative incidence of long Covid during the first year after infection was estimated to be 10 events per 100 persons in the pre delta period, 7.8 events per 100 persons in the omicron period, and about 3.5 events per 100 persons during the omicron era in vaccinated individuals (2). Noteworthy, this incidence decrease was actually independent of SARS-CoV-2 genetic variant since it was also observed between the first and second epidemic waves that involved the same variant in 2020 (3). However, prevalence estimations may vary greatly depending on the definition used (4). Indeed, although the main symptoms of long COVID reported by patients and the literature are fatigue, respiratory disturbances and cognitive issues (such as “brain fog”), a multitude of unspecific symptoms has been reported (5). According to the WHO definition established by Delphi method, there is neither a maximum timeframe for its onset after COVID infection—although it is stated that symptoms “usually occur 3 months from the onset of COVID-19” —nor a necessity for proof of SARS-CoV-2 infection to retain the diagnosis (6). Therefore, any unexplained symptom that occurred after March 2020 and lasted more than 2 months potentially meets the definition of long COVID.

## A critical approach to literature

A wealth of medical literature has developed since the summer of 2020 regarding the potential causes of long COVID, which is particularly difficult to synthesize due to significant heterogeneity. This might partly be due to publication bias and frequent methodological flaws.

First, as mentioned above, the lack of specificity of the long COVID definition allows very dissimilar populations to be included in studies. The early studies primarily included patients who had been hospitalized for severe COVID (7). These patients often had objective pulmonary sequelae and non-specific physical sequelae due to prolonged hospitalization (malnutrition, muscle atrophy, post-traumatic stress…). Then, studies tended to mix this population and patients who were not hospitalized for COVID-19, some of whom did not even have confirmed SARS-CoV-2 infection due to the lack of availability of testing in the community during the early months of the epidemic (5). It is this second population that poses a real problem due to the absence of an obvious cause for symptoms that can nevertheless be severe and very disabling. Unlike the post-hospitalization population, the majority are women, with an average age between 30 and 50 years (compared to over 60 years for hospitalized patients) and few comorbidities. Unfortunately, most translational studies on long COVID do not describe how patients were recruited, nor their clinical characteristics, and do not adjust their statistical analyses for the presence of comorbidities, even though these could explain part of the results (8–10). The early immunological studies also did not include an appropriate control group: they compared healthy subjects who had never been infected with COVID to patients with long COVID and found higher level of inflammation in patients (11), while it is now well established that sub-clinical inflammation markers decrease after infection but can persist in the human body for several months, independently of the persistence of symptoms (10, 12, 13). Therefore, the appropriate control group is patients who were infected by Sars-Cov-2, but did not have persistent symptoms with the same follow-up time since infection than patients with long COVID. Furthermore, immunological studies involve numerous cytokine assays, cellular phenotyping, transcriptome studies, etc., using modern multiplex methods, but very few consider the alpha risk inflation (false positive results) due to the multiplication of statistical tests. Additionally, most of them only highlight positive results and fail to discuss negative findings that contradict other publications (8–12). Finally, even in the case of statistically significant differences, the distributions of biological marker concentrations largely overlap between cases and controls, preventing their use as prognostic or diagnostic markers. Thus, although many immunological markers have been shown to be marginally but differentially distributed between cases and controls, none have been consistently replicated to date (10–12, 14). Consequently, no consensus can be reached regarding the potential specific immunological mechanisms at play in the genesis of long COVID (15).

Similarly, early studies exploring viral persistence were conducted without controls or with inappropriate controls (patients who had never been infected) or at an early stage (less than 3 months symptoms duration). Some of them suggested that viral persistence could explain long COVID based on the presence of SARS-CoV-2 RNA in olfactive bulbs, digestive biopsies or feces (16). However, subsequent studies including patients with or without persistent symptoms after COVID-19 did not find evidence of longer viral persistence in those with persistent symptoms (12, 17). One recent study investigated the persistence of viral RNA in various tissue samples of patients who had mild COVID-19. A significant association has been identified between the detection of viral RNA in at least one tissue and the presence of long COVID symptoms. This association strongly decreased between 1 and 2 months after infection and was no more significant 4 months after infection (18).

Regarding the specific aspect of central nervous system (CNS) involvement, it is important to note that persistence of SARS-CoV-2 in the CNS has never been directly described in long COVID. Studies suggesting the presence of SARS-Cov-2 in the brain have been conducted using autopsies of patients who died of severe acute COVID-19. They found CNS symptoms such as hemorrhagic infarction, microglial activation and neuronal phagocytosis, but detectable levels of virus in the brain were very low and not associated with histopathological changes (19). Furthermore, while *in vitro* studies have suggested several theoretical pathways by which this virus may enter the CNS, clinical studies suggest that direct invasion of the CNS by SARS-CoV-2 is rare and extremely limited. Nevertheless, it is possible that the SARS-CoV-2 spike (S) protein has direct inflammatory and procoagulant effects. The addition of cytokine release syndrome (CRS) with loss of blood–brain barrier integrity may contribute to the expression of pro-inflammatory mediators by neural cells that may affect brain function (20). However, as the presence of Sars-Cov-2 RNA in other tissues, markers of CNS damage do not correlate with long-term clinical symptoms. For example, a study comparing the CNS effects of the virus during the acute phase of COVID-19 and six months later found that plasma concentrations of neurofilament light chain (sNfl) and glial fibrillary acidic protein (GFAp) normalized, while a large number of patients continued to have neurological and cognitive symptoms (21). A notable exception may be noted for persistent anosmia/ageusia, which correlates with evidence of viral material and inflammation in olfactive bulbs/tongue biopsies (22, 23). It should also be noted that prolonged viral persistence has been well (and easily) documented in patients who are severely immunocompromised (notably organ transplant recipients), who also have a very different clinical presentation and evident paraclinical anomalies (18, 24). In contrast to studies using ultra-sensitive biological techniques not commonly used in current practice (mostly dosage of plasma cytokines by multiplex essay or leukocyte immunophenotyping by flow cytometry), studies published by clinicians consistently failed to demonstrate biological difference between patients infected by COVID-19, with and without persistent symptoms (17, 25–30).

Last, 4 years after the clinical characterization of long COVID, no efficient pharmacological treatment has been reported (31, 32). In particular, unlike in acute COVID-19, neither antiviral drugs, anti-SARS-CoV-2 monoclonal antibodies, nor immunosuppressive drugs such as steroids or interleukine-6 inhibitor have proved efficient in long COVID (33, 34).

By contrast, certain non-biological risk factors have been regularly identified in patients with long COVID and mild initial COVID-19, such as female sex and the number of initial symptoms (29, 35), the history of anxiety or depressive disorders (28, 36–39), or negative feelings regarding COVID-19, such as the COVID-related anxiety (40), the burden associated with symptoms of acute COVID (41), and the fear that acute symptoms will persist (42). It is unfortunate that this non-somatic dimension is completely ignored, even in the most recent high-quality reviews of the causes of long COVID (1, 43).

## Arguments for a functional disorder

For patients who search for information on the internet, or for doctors who are not expert clinicians, the combination of long COVID symptoms may evoke several rare pathologies: systemic immunological diseases (lupus, vasculitis, connective tissue diseases, autoinflammatory diseases, etc.), hematological conditions (mast cell activation syndrome…), infectious or genetic diseases (cryopyrinopathies, interferonopathies…). However, unlike patients suffering from these biologically explained diseases, patients with long COVID do not present objective clinical signs that would allow their diagnosis. Most symptoms are either subjective or compatible with a dysfunction of the autonomic nervous system (hyperventilation, postural orthostatic tachycardia syndrome…), but without criteria of severe dysautonomia (38, 44). Furthermore, in patients without history of severe acute COVID-19, there is no abnormal biological or imaging findings or they cannot entirely explain the symptoms (13, 17, 26–30). As mentioned above, one exception is anosmia and dysgeusia, that are associated with pathological findings at MRI and nose or tongue biopsies (23, 45) and probably arise from direct neurological viral toxicity. For other symptoms than anosmia and dysgeusia, the only abnormal results that are frequently observed are hypometabolisms of right medial temporal lobes (hippocampus and amygdala), right thalamus brainstem and cerebellum at brain PET scans (46), whose interpretation is controversial. Indeed, there is no established correlation with the type and intensity of symptoms (47) and the cause of the observed anomalies could be organic or functional (48).

The clinical picture of long COVID, on the other hand, has strong semiological similarities with other biomedically unexplained conditions that have different presumed causes (like chronic Lyme disease, hypersensitivity to electromagnetic waves or chemicals, etc.) or are defined by a main symptom (fatigue for myalgic encephalomyelitis/chronic fatigue syndrome, pain for fibromyalgia, etc.). It is commonly, though not unanimously accepted, that these entities are part of the broader group of “functional somatic disorders” (FSD) (49). FSD are usually defined as patterns of persistent bodily complaints for which adequate examination does not reveal sufficiently explanatory structural abnormality or other specified pathology, with severe impact on functioning and quality of life (49–51). FSD vary in names based on the predominant symptoms and the medical specialty involved (e.g., irritable bowel syndrome in gastroenterology, hyperventilation syndrome in pneumology, fibromyalgia in rheumatology, chronic fatigue syndrome in internal medicine….). They represent the medical side of the psychiatric nosologic category “somatic symptom disorder” in DSM V (50). Importantly, FSD is often triggered by a somatic illness (in particular an infectious disease) but also involves brain conditioning along with socio-psychological predisposing factors (perfectionism, alexithymia, childhood traumatic experience…). Most importantly the long term persistence of symptoms is favored by cognitive (involuntary attentional focusing on symptoms, catastrophism, illness-related anxiety, feeling of rejection…) and behavioral factors, including avoidance of physical effort that leads to physical deconditioning as well as avoidance of uncertainty that leads to never-ending request for medical tests and consultations (51, 52). These conditions can be associated to varying degrees in the same person, suggesting shared transdiagnostic mechanisms (49, 51), Thus, the term “bodily distress syndrome” (International Classification of Diseases 11), has been suggested as a more neutral term to cover them all (53). Strikingly, bodily distress syndrome shares all its symptoms with those that are most common in long COVID (see Table 1).

**Table 1:** Diagnostic criteria for bodily distress symptoms.

1. ≥ 3 symptoms from at least one of the following groups: *Cardiopulmonary/autonomic arousal:* Palpitations /heart pounding, precordial discomfort, breathlessness without exertion, hyperventilation, hot or cold sweats, dry mouth *Gastrointestinal arousal:* Abdominal pains, frequent loose bowel movements, feeling bloated/full of gas/distended, regurgitations, diarrhea, nausea, burning sensation in chest or epigastrium *Musculoskeletal tension:* Pains in arms or legs, muscular aches or pains, pains in the joints, feelings of paresis or localized weakness, back ache, pain moving from one place to another, unpleasant numbness or tingling sensations *General symptoms:* Concentration difficulties, impairment of memory, excessive fatigue, headache, dizziness. 2. The patient has been disabled by the symptoms (i.e., daily living is affected) 3. Relevant differential diagnoses have been ruled out

A significant number of symptoms observed in patients with long COVID are also similar to those found in people suffering from post-traumatic stress disorder (PTSD). In particular, experiencing neurocognitive symptoms, such as difficulties with memory and thinking, after mild COVID-19 infection was strongly associated with the presence of persistent PTSD-like symptoms (54). The occurrence of PTSD is common in the context of infectious epidemics (55) Noteworthy, in contrast to patients with FSD, patients with PTSD experience flashbacks—reliving the traumatic COVID episode, or have recurring memories or dreams related to this acute COVID episode. Therefore, this condition is essentially observed in patients who have dealt with severe COVID-19 (56).

In our clinical center dedicated to long COVID in Paris, after standardized multidisciplinary evaluation, 76% of patients who had mild acute COVID-19 and exhibited prolonged symptoms (median duration 429 days) meet the criteria for FSD (57). This observation has been shared by other clinicians worldwide (58–60). In our experience, 21% patients were also diagnosed with (i) anxiety (including panic disorders, whose manifestations are primarily physical) or (ii) depressive disorders that account for their symptoms, (iii) with or without associated FSD. This is consistent with a recent meta-analysis reporting a global prevalence of depression and anxiety in 23% of patients with long COVID (61). In our cohort, only a minority of patients (10%) did not fit into one or more of these three diagnoses, most of them having another condition explaining the symptoms, unlinked to COVID-19 (57).

A final argument in favor of the FSD hypothesis is that to date, only cognitive behavioral therapy and gradual physical activity have proven effective in treating long COVID (34). It is noteworthy that, whereas nirmarelvir/ritonavir was reported as inefficient as a curative for long COVID (33), it has been successfully tested as a preventive treatment (62). This finding is not surprising even in the hypothesis of FSD, as nirmarelvir/ritonavir decreases the intensity and the number of symptoms of the acute episode of SARS-CoV-2, which are risk factors for long COVID.

## Current management of patients with long COVID symptoms

In Belgium, the management of long-COVID is primarily predicated on a personalized care pathway, which is funded by the Ministry of Health. As also recommended in French national guidelines (63), this care is coordinated by the general practitioner, who refers patients to various health professionals, including physiotherapists, ergotherapists, neuropsychologists, and dieticians.

Unfortunately, many physicians are reluctant to handle these patients, who often require considerable time and attention, leading to diagnostic and therapeutic failures. Many tend to rid themselves of the problem either by dismissing the legitimacy of the complaint (“you do not have anything!” or “it will pass on its own!”), or, on the contrary, by conducting numerous tests and requesting many specialized opinions to reduce their own uncertainty, out of fear of missing a serious illness (64). This second attitude is understandable to the novelty of COVID-19 infection, its pleiomorphism and sometimes alarming scientific literature. However, this diagnostic quest quickly becomes detrimental for the patients. Both attitudes aggravate the situation, with the first intensifying feelings of rejection and the second exacerbating catastrophizing, both worsening the attentional focus on symptoms, which perpetuates or even exacerbates symptoms. In the doctors’ defense, an exhaustive search—though impossible—is often advocated by the patients themselves.

Indeed, patients with FSD spontaneously consult doctors because of the physical nature of their symptoms and are generally reluctant to be referred to mental health specialists. Even when patients do accept a psychiatric assessment, the psychiatrist most often focuses on the identification and treatment of classical psychiatric disorders (anxiety, depression, etc.), which affect only a minority of patients. Few psychiatrists are trained to actively seek out FSD and fear misattributing physical symptoms to a psychological cause. A “return to sender” is therefore common, further reinforcing the patient’s belief in an exclusively somatic cause (51, 64). This path marked by non-recognition and medical nomadism is that of patients with long COVID and is an integral part of their problem. In fact, in most countries, the notion of FSD is very poorly understood and is often equated with a psychiatric illness, or attributed exclusively to the patient, or at worst, seen as malingering. This leads to significant hetero and self-stigmatization, as well as a feeling of non-recognition or even humiliation, which perpetuates the need to prove the reality of the symptoms and to search for an external, or at least physical, cause.

There is indeed a major training deficit for FSD in somatic physicians, psychiatrists, psychologists, physiotherapists, and the general population. One of the problems is the poor reputation of psychiatric illnesses and the belief in a body/mind duality, which often leads to the rejection of any “psychologizing” explanation. Recently, a German team proposed a very integrative vision of persistent physical symptoms (PPS)—that is, symptoms lasting several months, regardless of their cause (52)—which seems to particularly apply to long COVID. These symptoms affect up to 9% of the general population. The more they persist, the more their link to a pathophysiological cause weakens. Examples include persistent digestive symptoms after the remission of chronic intestinal disease, hyperventilation syndrome distinct from co-existing asthma, or chronic fatigue syndrome following a viral infection. The factors of chronicity are biological (e.g., low-grade inflammation, alterations in microbiome…), cognitive-perceptual and emotional (e.g., symptom focus, catastrophism, alexithymia, health-centered anxiety), behavioral (e.g., physical deconditioning due to inactivity and avoidance behaviors), and related to interaction with the health system (e.g., drug side effects and conflicting relationships with health care professionals). There is a continuum between physical and psychological causes, but even diseases with a well-accepted pathophysiological explanation, such as systemic lupus erythematous, multiple sclerosis or spondyloarthropathy, are strongly modulated by cognitive-behavioral factors. When there is a discrepancy between a high symptom burden and normal clinical and paraclinical exams, these PPS meet the criteria for FSD.

We believe that this vision is capable of reconciling patients and doctors, based on a shared and accepted diagnosis. It allows them to focus on the essential, which is the personalized search for effective therapeutic solutions. In our experience and that of many colleagues, acceptance of the diagnosis is very good if it is explained in a positive and scientific way, with empathy and without judgment (65). This includes providing rational cognitive explanations for the symptoms and support for behavioral changes, such as stopping medical explorations and resuming exercise very gradually.

## What place for the general internist in the management of patients with long-COVID?

Because of the multiplicity of possible causes, internists have several assets that should in theory allow them to take good care of patients with long COVID who do not improve after a first line treatment by their general practitioner. First, they have large semiology skills and good knowledge of the potential differential diagnoses (including immunological, metabolic and multi-system infectious diseases) that should allow them to avoid unnecessary and deleterious examinations if the clinical presentation is incompatible.

In France, the post graduate teaching of internal medicine is coupled with that of clinical immunology for 5 years. Unfortunately, French internists are also often guilty of excessive diagnostic work-up, which is sometimes poorly related to symptoms. However, they are used to coordinating care with other specialists, thus avoiding or at least reducing medical nomadism. They also usually have a genuine willingness to achieve holistic care. Finally, they have a long-standing experience with patients with unexplained symptoms, including many patients with FSD who consult them in the hope for a new diagnostic.

**Figure 1:**
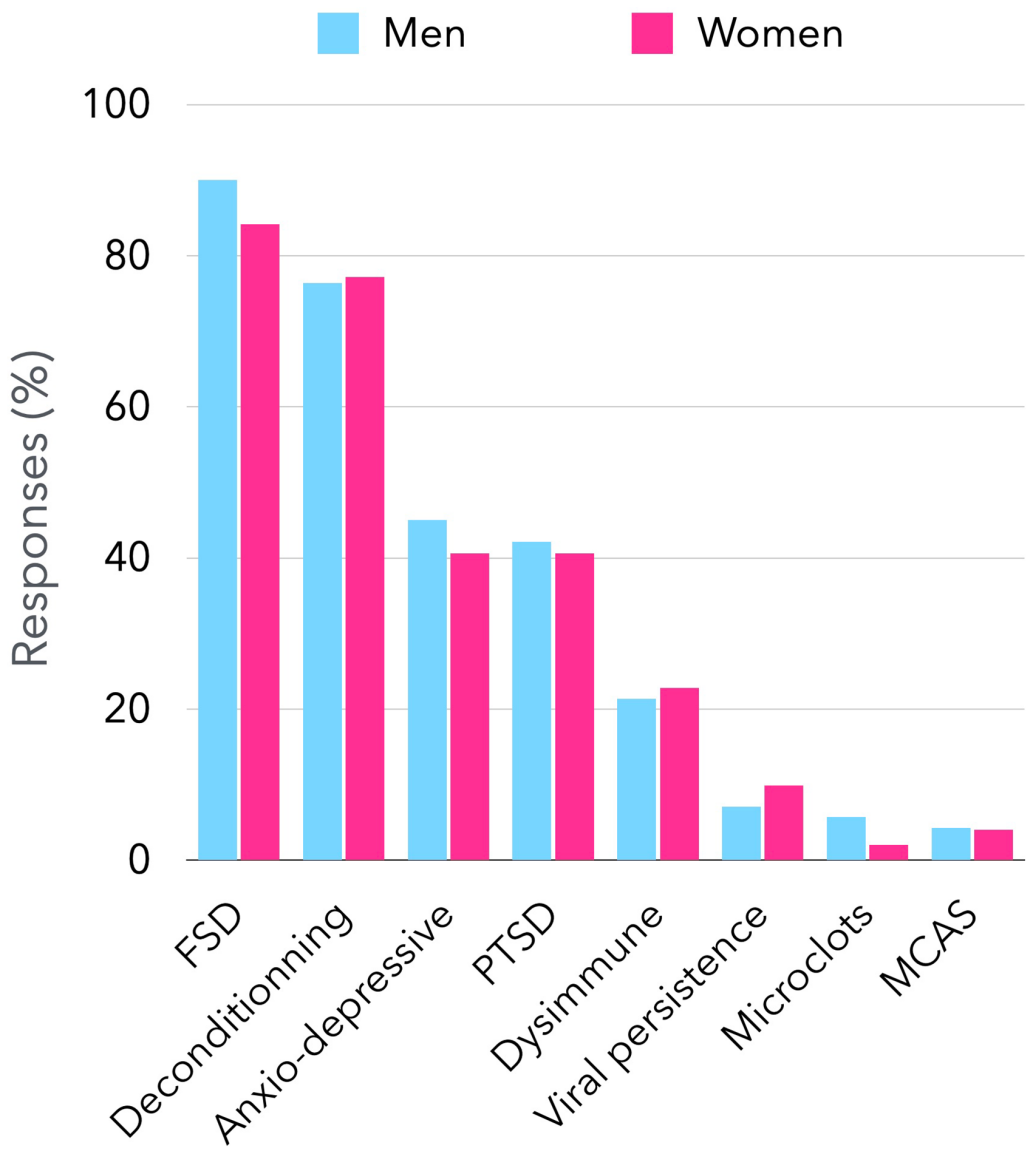
Possible causes for long COVID considered by senior internists in France. The figure presents the findings of a survey conducted among senior internists who are members of the French Society of Internal Medicine regarding the causes of long-term symptoms associated with long COVID. By the close of March 2025, a total of 240 responses had been documented through a Google Form platform, accessible via an access link. Participants were invited to identify one or more possible causes of long COVID, which are shown on the x-axis. The figure illustrates the proportion of respondents (both male and female) who consider each of the eight propositions. There was no significant difference between men’s and women’s responses. FSD: functional somatic disorder. PTSD: post-traumatic stress disorder. MCAS: mast cell activation syndrome.

**Figure 2:**
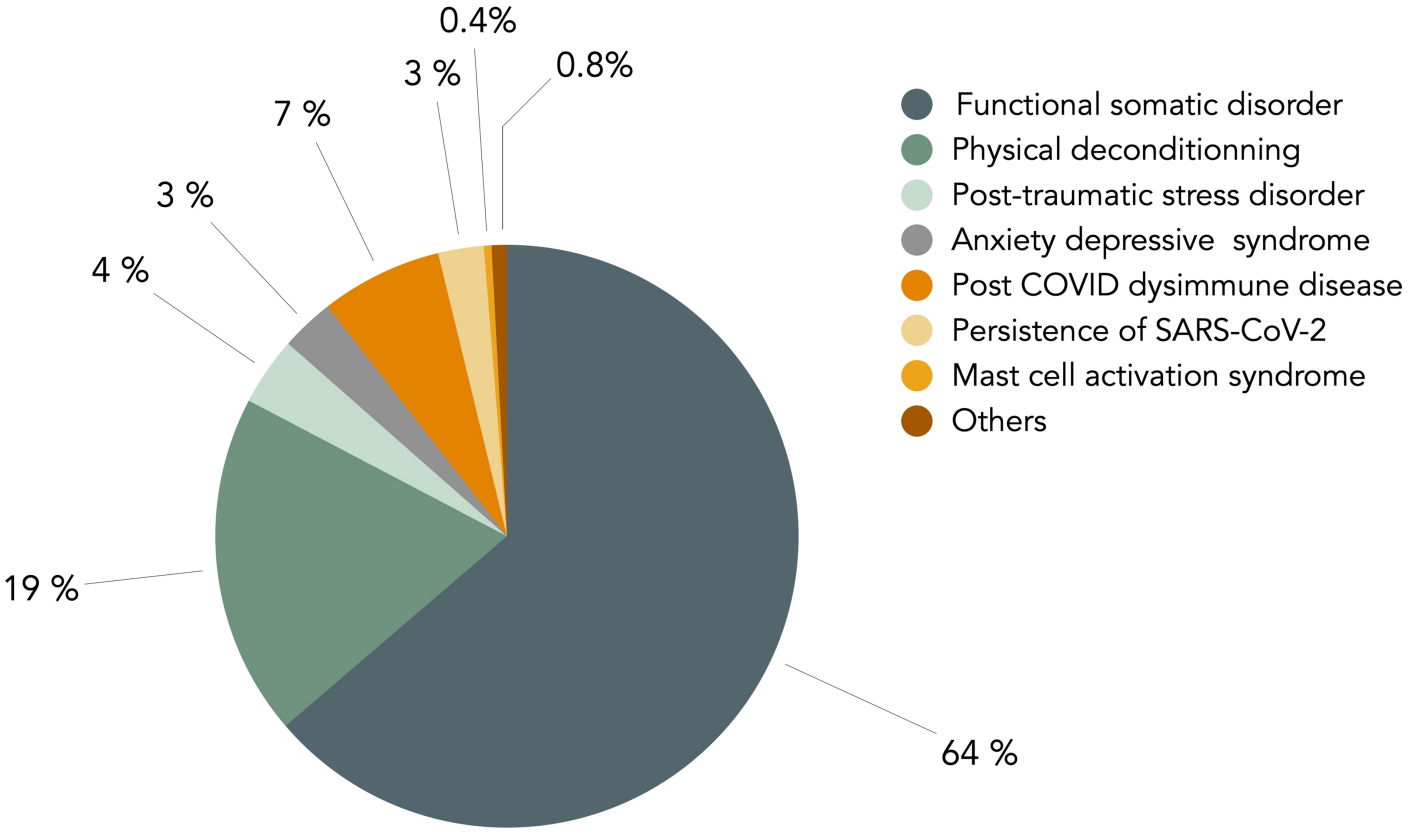
Main cause for long COVID considered by senior internists in France. In the survey delineated in the legend of Figure 1, a second question was asked of senior French internists about the main cause of long COVID. The figure presents a graphical representation of the responses to this question. The question posed to participants was: “Among the hypotheses you selected in the initial question, which do you consider to be the primary cause?” The non-somatic causes, depicted in grey-green, account for a substantial proportion of the responses, amounting to 90%.

However, internists’ views on long COVID are far from unanimous. Recently, we performed an online survey of French senior internists, that showed that beliefs are disparate. Among 240 responders (females 42%), representing all French regions, age groups and type of medical practice (Supplementary Figures 1, 2), 214 (89%) considered that long COVID may be an FSD. They also think other causes may be associated, such as physical deconditioning (77%), post-traumatic stress (41%), anxio-depressive disorder (43%), dysimmune disease (23%), SARS-CoV-2 persistence (8%) or other miscellaneous hypotheses (Figure 1). When they were asked to choose a primary cause, 63% chose FSD, 19% physical deconditioning, and only 9% biological cause (Figure 2).

One striking fact is that 229/240 (95%) of French internists do not want to manage patients with long COVID on their own, contrary to most multi-systemic immune mediated inflammatory disorders. Many internists (111/240, 46%) do not want to take care of them anymore once the etiological assessment is carried out and does not highlight any objective anomaly. Even more, 69 (29%) wish they would not see any patient with long COVID in consultation. This is a good example of the rejection experienced by patients with FSD, which makes their fear of being labeled with this diagnosis quite understandable. This is partly due to a lack of doctors’ training for FSD diagnostic and treatment. Indeed, most internists, although they often quickly have the intuition that the patient has a FSD, are not aware of their specific positive criteria. Therefore, they usually retain this diagnosis by default, after a very broad biomedical work-up and often without telling the patient explicitly. Furthermore, even with a good knowledge of FSD, consultations are often difficult and sometimes frankly tedious for the doctor. Notably, the time of anamnesis is particularly long (easily an hour if one tries to be exhaustive) and difficult to synthesize afterwards. The mobilization of empathy must be maximum and requires a lot of energy. Last, the management of uncertainty is anxiogenic (“Doctor, how can you be sure that you have looked for everything?”). Thus, these consultations are very energy and time consuming, and most physicians fear them (66).

Therefore, almost all French internists endorse a multidisciplinary management of patients with long COVID, as they do for patients with FSD. Noteworthy, existing national management guidelines for long COVID (63, 67) also praise for such a holistic approach, modeled on existing recommendations for FSD (65, 68), even if FSD is not mentioned explicitly or even excluded (69). Indeed, such an approach is recommended for several other complex conditions without any detectable organic lesion, such as fibromyalgia (70) or chronic fatigue syndrome (71). Both graduated physical activity (72) and cognitive behavioral therapy (73, 74) proved efficient in individuals with long COVID. Physical rehabilitation is usually well tolerated if well explained and realized correctly (75). However, if the exercise intensity is initially too high, the occurrence of post exertional malaises can reinforce the fear of exercising. Although no trial has assessed the superiority of a multidisciplinary approach combining graduated physical activity and cognitive behavioral therapy, trials are ongoing (e.g., ECHAP COVID, https://clinicaltrials.gov/study/NCT05532904) and integrated programs have already provided high satisfaction rates among patients with severe long COVID (57). Such programs include the delivery of rational cognitive explanations for the symptoms and support for behavioral changes tailored to the patient. Many patients with very disabling long COVID, who benefited from this type of psycho-corporal treatment, have also reported their recovery story on the Norwegian site recoverynorway.org.

## Conclusion

Along with Saunders et al. (76) we think that “it is time to break taboos based on a dualistic understanding of physical versus mental illness and bring in existing knowledge about functional somatic symptoms to provide improved explanations and treatments.”

Except for those patients who have an identified cause of prolonged symptoms, such as depression or PTSD or post intensive care physical sequelae, we argue to treat long COVID as a FSD, rather than waiting for hypothetical pharmacological treatments that biological studies might bring us in the future. It is therefore necessary to federate motivated and competent health care professionals to distribute the mainstays of treatment in a coordinated and synergistic way (77). In addition to the physicians, several other health care professionals are key actors of the patient’s recovery, such as psychiatrists, psychologists, physiotherapists, speech-language pathologists and teachers of adapted physical activity.

The position of the physician must probably remain central due to the physical nature of long COVID symptoms, with regular reassessment to not omit another associated disease. This is crucial to reassure the patient, so that he/she can concentrate on his/her personal physical and mental work. Along with general practitioners, internists certainly have a key role to play in the management of patients with the most severe conditions. Nevertheless, all health care professionals certainly need to be trained to better know the various mechanisms in play in the persistence of symptoms, avoid inappropriate behaviors and communication mistakes (78) and tailor patient-centered appropriate management, especially regarding the modalities of resuming physical activity. To be widely accepted, this proposal requires a radical change in the way mind–body interaction is viewed in the medical community and the general population. Less alarmist and more balanced media coverage should help the public to recognize the reality of FSD, understand its mechanisms and the potential for complete recovery.

## Supplementary material

SUPPLEMENTARY FIGURE 1 Demographics and practice patterns. The results of the demographic data and professional practice of 240 French internists who responded to our survey on long COVID are presented. This sample reflects the practice of internal medicine in France, with a higher proportion of men **(A)**, an age distribution with most internists aged between 40 and 49 **(B)**, and most of the practice in university or general hospital structures **(C)**. The distribution of the number of long COVID patients treated by the respondents is shown in **(D)**. A very small proportion of respondents do not treat any patients and more than one in three treat more than 50 patients. ESPIC, Private Health Establishment of Collective Interest.

SUPPLEMENTARY FIGURE 2 Geographical distribution of survey respondents. The figure shows the geographical location of internists who responded to the long COVID survey. This distribution is consistent with the distribution of internists by region in France.
