# Supplementary figures and images for: Internal medicine at the crossroads of long COVID diagnosis and management
Source: Front Med (Lausanne). 2025 May 2;12:1521472. doi: 10.3389/fmed.2025.1521472 (PMC12083770; doi:10.3389/fmed.2025.1521472)

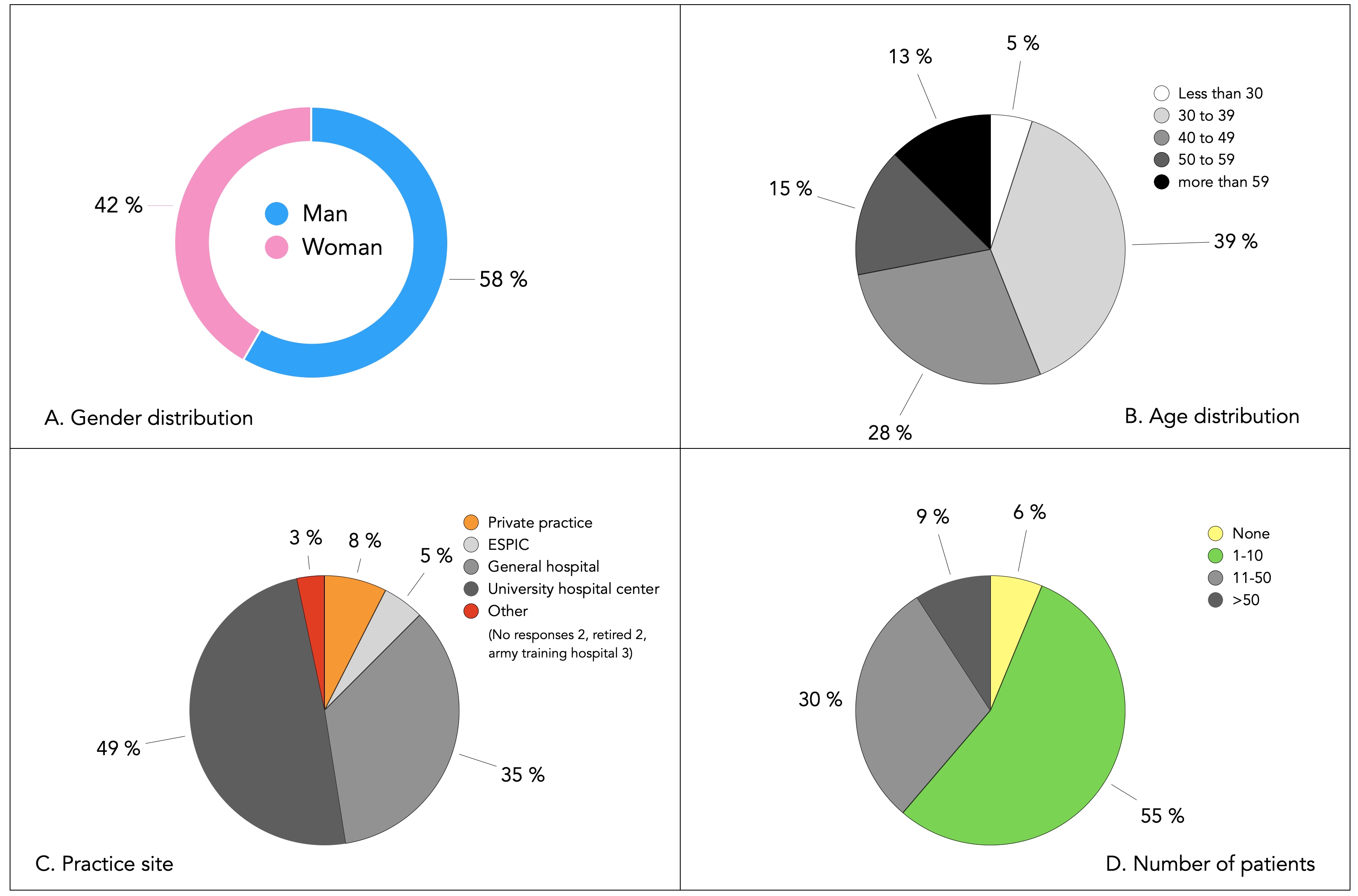

Supplement: SUPPLEMENTARY FIGURE 1 — Demographics and practice patterns. The results of the demographic data and professional practice of 240 French internists who responded to our survey on long COVID are presented. This sample reflects the practice of internal medicine in France, with a higher proportion of men (A), an age distribution with most internists aged between 40 and 49 (B), and most of the practice in university or general hospital structures (C). The distribution of the number of long COVID patients treated by the respondents is shown in (D). A very small proportion of respondents do not treat any patients and more than one in three treat more than 50 patients. ESPIC, Private Health Establishment of Collective Interest. [file Image_1.jpeg]

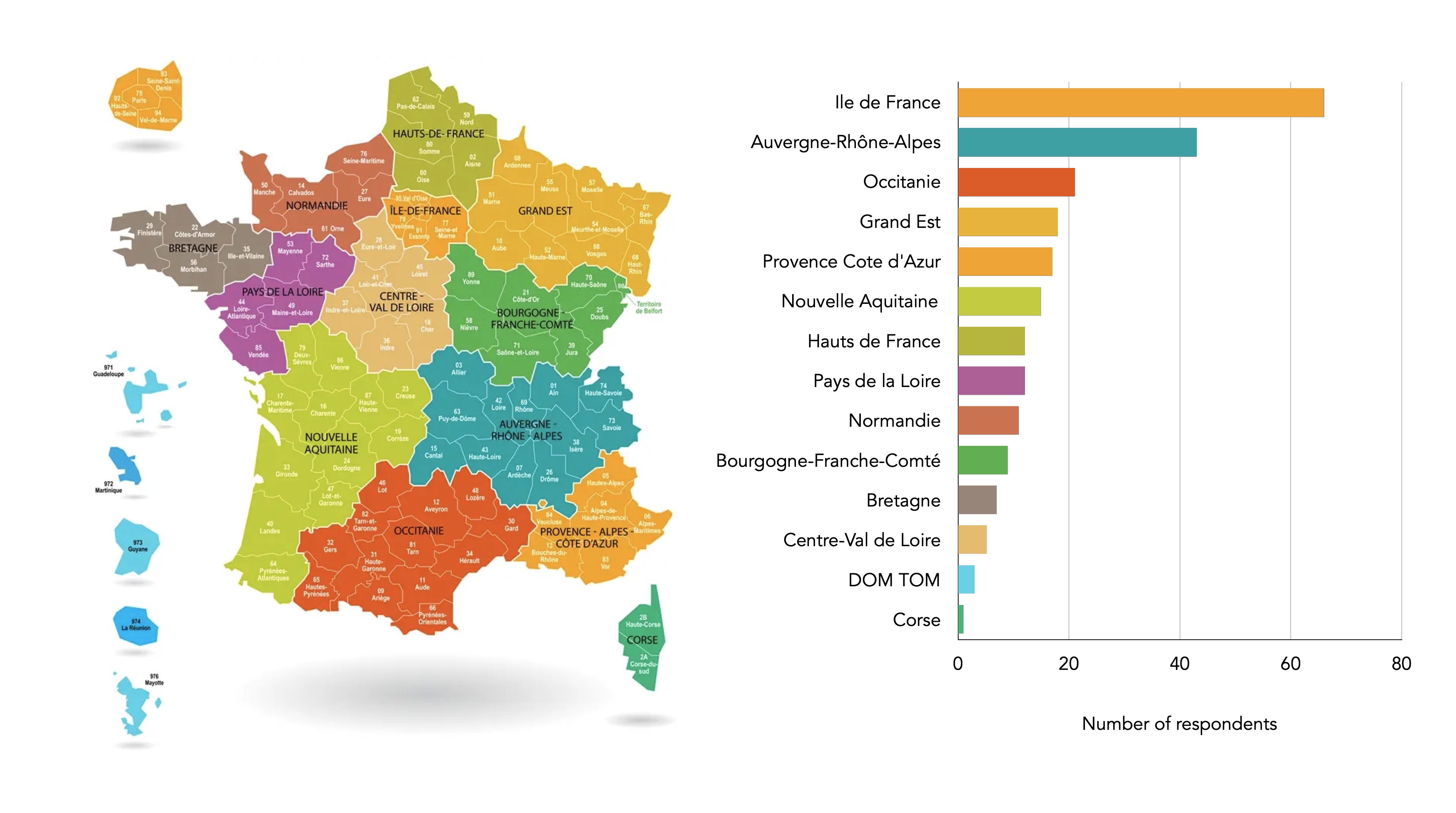

Supplement: SUPPLEMENTARY FIGURE 2 — Geographical distribution of survey respondents. The figure shows the geographical location of internists who responded to the long COVID survey. This distribution is consistent with the distribution of internists by region in France. [file Image_2.jpeg]
